# Evaluation of Dual-Cure Resin Cement Polymerization under Different Types and Thicknesses of Monolithic Zirconia

**DOI:** 10.1155/2019/4567854

**Published:** 2019-01-09

**Authors:** Pinar Turkoglu, Deniz Sen

**Affiliations:** ^1^DDS PhD, Istanbul University Faculty of Dentistry, Department of Prosthodontics, Istanbul, Turkey; ^2^Professor, Istanbul University Faculty of Dentistry, Department of Prosthodontics, Istanbul, Turkey

## Abstract

**Purpose:**

The aim of the present study was to investigate the effects of the type and thickness of the zirconia material on the microhardness of the underlying dual-cure resin cement.

**Materials and Methods:**

Eight disk-shaped zirconia specimens with a 4-mm diameter and four varying thicknesses (0.5, 1.0, 1.5, and 2.0 mm) were fabricated from two different monolithic zirconia materials: posterior monolithic zirconia (Prettau) and anterior monolithic zirconia (Prettau Anterior). Dual-cure resin cement specimens with a 4-mm diameter and 5-mm height were prepared using Teflon cylinder molds and activated by light beneath the eight zirconia disks and without any zirconia disk for 20 s (n=12). A total of 108 specimens were embedded in acrylic. Vickers hardness of each specimen was measured at three different depths using a microhardness device with a 50-g load. All data were statistically evaluated using three-way ANOVA, one-way ANOVA, independent samples t-tests, and Bonferroni corrected post hoc tests (*α*=.05).

**Results:**

Dual-cure resin cement's microhardness was significantly higher for the samples polymerized beneath anterior monolithic zirconia compared to posterior monolithic zirconia. The hardness decreased as the thickness increased for both types of zirconia; the latter was attributed to an attenuated curing efficiency.

**Conclusion:**

Microhardness of the dual-cure resin cement is influenced by both the type and the thickness of the monolithic zirconia restoration. Polymerization efficiency for resin cement cured under anterior monolithic zirconia may be superior to cured beneath posterior monolithic zirconia.

## 1. Introduction

Densely sintered yttria-stabilized zirconia ceramics (Y-TZP) have been widely used as alternatives to metal-ceramic restorations because of their superb mechanical properties and favorable esthetic characteristics [1, 2]. They can be fabricated using either computer-aided design/computer-aided manufacturing (CAD/CAM) technology or manually controlled copy-milling techniques [3, 4]. However, light transmission through zirconia-based restorations is critical, and the translucency of these restorations has been found to be less than that of glass ceramics because of the high crystalline content of zirconia [5, 6]. For this reason, after milling, zirconia cores have to be veneered with porcelain using a layering technique in order to overcome the esthetic problems associated with inadequate translucency of the material. However, these porcelain veneers exhibit a lower strength than zirconia, resulting in chipping and cracking problems during chewing [7]. To overcome this problem, single-layer zirconia restorations fabricated from translucent zirconia blocks with full anatomical contours have recently become available as an alternative to bilayered restorations for dental applications [8]. These monolithic zirconia restorations exhibit a somewhat higher resistance to chipping and/or cracking than do layered zirconia restorations; moreover, their esthetic properties are superior because of their greater translucency [9]. Prettau® Anterior is a monolithic Y-TZP material with high translucency that has been improved for use in the anterior tooth region as an alternative to lithium disilicate ceramic. It is fabricated with an increased yttria content (>8 mol%) in order to achieve full stabilization and improved translucency due to the increased cubic phase [10, 11].

Either type of zirconia-based restorations can be cemented using either conventional or adhesive techniques [12]. Adhesive cementation with a phosphate monomer resin cement is preferred for zirconia-based restorations because of low solubility, enhanced esthetic properties, and high bond strengths, particularly in case of insufficient retention and resistance forms [12]. However, several studies have reported that dual-cure resin cements need sufficient light polymerization to achieve the required mechanical properties. In other words, the chemical polymerization constituent of dual-cure resin cement cannot compensate for the lack of light polymerization [13–15]. Therefore, the amount of light passing through a restorative material should be sufficient enough to promote the polymerization process, even though the materials undergo both chemical and light polymerization [16].

Various novel monolithic zirconia materials with different compositions and relatedly translucencies are available in the dental market. However, the effect of their type and thickness on the polymerization competence of resin-based cements remains to be a subject of interest. Therefore, the purpose of the current study was to investigate the microhardness of dual-cure resin cement used underneath anterior and posterior monolithic zirconia materials with different thicknesses. The null hypothesis of the study was that the microhardness of dual-cure resin cement is not affected by the type and the thickness of the overlying monolithic zirconia material.

## 2. Materials and Methods

### 2.1. Zirconia Disk Preparation

Eight disks with a 4-mm diameter and varying thicknesses were prepared from two types of monolithic zirconia materials (Table 1); four PRZ disks were prepared from presintered partially stabilized white zirconia blocks (Prettau Zirconia, Zirkonzahn GmbH, Bruneck, Italy) whereas four PRA disks were fabricated from fully stabilized white zirconia blocks (Prettau® Anterior, Zirkonzahn GmbH) using a CAD/CAM system (Zirkonzahn GmbH). The thicknesses of the disk samples were 0.5, 1.0, 1.5, and 2.0 mm (Table 2). The PRZ disks were immersed in coloring liquid with the A2 shade (Color Liquid for Prettau, Zirkonzahn GmbH) using plastic pliers. After 3 s, they were removed and allowed to dry under a warming lamp (Zirkonlampe 250, Zirkonzahn GmbH) for 30 min in accordance with the manufacturer's recommended time. The colored samples were sintered in a sintering oven (Keramikofen 1500, Zirkonzahn GmbH), with a temperature increase from 20°C to 1500°C over 3 h; the samples were kept at 1500°C for 2 h. The PRA samples were stained with the A2 shade Color Liquid (Prettau® Anterior Aquarell, Zirkonzahn GmbH). Two coats of staining liquid were applied with a brush, and the samples were placed for 20 min under an infrared drying lamp (Zirkonlampe 250, Zirkonzahn GmbH) according to the manufacturer's instructions. The stained disks were sintered in a furnace (Zirkonofen 600/V2, Zirkonzahn GmbH) that was initially at room temperature; the temperature was increased to 1450°C at the rate of 5°C/min. The samples were kept at 1450°C for 2 h, followed by cooling to room temperature at the rate of 5°C/min.

The total thickness of all zirconia disks was measured by a digital caliper. Then, the outer disk surfaces were glazed (Glaze Plus; Zirkonzahn GmbH) in accordance with the manufacturer's directions.

### 2.2. Resin Cement Sample Preparation

**Figure 1:**
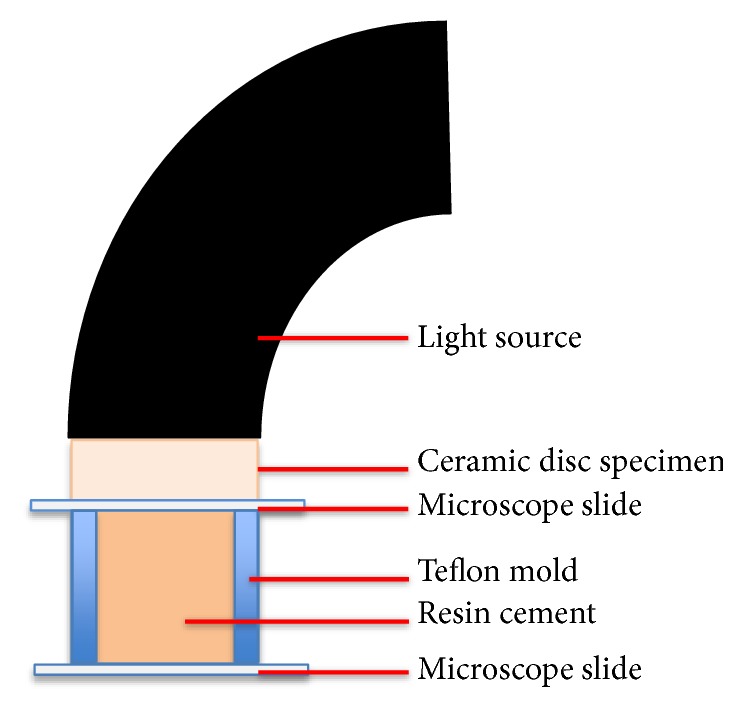
Schematic showing the method of preparation for dual-cure resin cement samples [17]. “Figure reproduced from Turp et al. (2018) [under the Creative Commons Attribution License/public domain]”.

In total, 108 resin samples were prepared by the placement of dual-cure resin cement (Panavia F 2.0 Light shade, Kuraray Medical, Inc., Osaka, Japan) in cylindrical polytetrafluoroethylene (PTFE) molds with standard dimensions (4.0-mm diameter and 5.0-mm height). For the preparation of resin specimens, a glass slide placed against a black background was used in order to support the surface and reduce the reflectance of the subjacent surface against each sample. A transparent film strip was placed on the glass slide to avoid bonding of the material. Equal amounts of base and catalyst were mixed according to the manufacturer's recommendations. The PTFE cylinder mold was filled with resin cement. One more transparent film strip was placed on top, followed by another microscope slide, using finger pressure. Excess cement material was removed from the mold by pressing the film strips between the glass slides. Following removal of the excess cement, one of the eight ceramic disks was placed on top, and the tip of the light source was smoothly located on top of the zirconia disk. This would allow light to pass through the disk over the material (Figure 1). Resin samples were randomly allocated to eight groups yielding 12 samples per zirconia disk (n = 12) and 12 control samples were prepared by direct light curing without any overlying monolithic zirconia. Thus, a total of 9 groups containing 12 specimens each were generated. An LED curing unit (Elipar S10, 3 M ESPE, Seefeld, Germany) with a wavelength of 430-480 nm and a power density of 1200 mW/cm^2^ was used with an exposure time of 20 s. Calibration of the curing device was checked before polymerization by contacting the tip of the light to a built-in light meter.

Subsequently, the resin cement samples were taken out of the PTFE molds, and a plastic spatula was used to remove the uncured material according to ISO 4049 guidelines [18]. The cured samples were kept in dry, light-proof containers for 24 h. Then, the microhardness of the resin was determined by calculating the Vickers Hardness Number (VHN) according to ISO 4049 guidelines [18].

### 2.3. Microhardness Test

**Figure 2:**
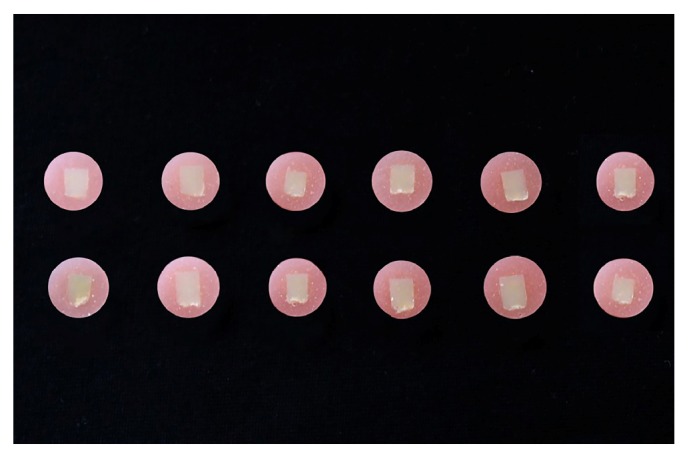
Dual-cure resin cement samples embedded longitudinally in acrylic following polymerization.

**Figure 3:**
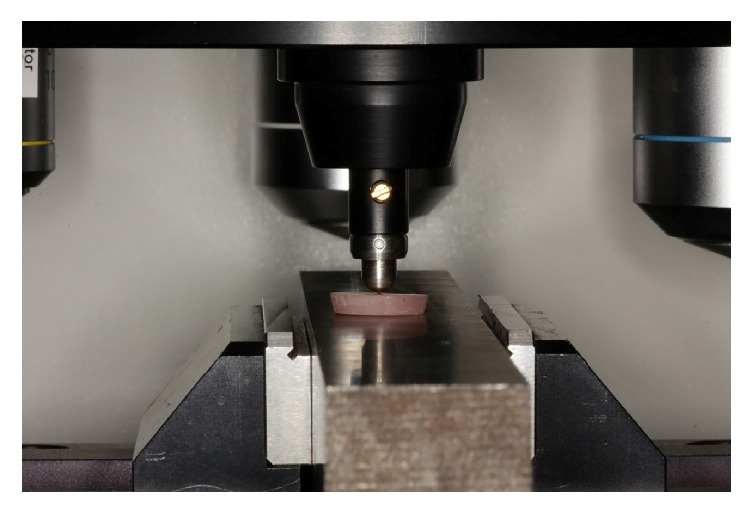
Vickers hardness indentation of a dual-cure resin specimen by the positioning knob of microhardness test device.

**Figure 4:**
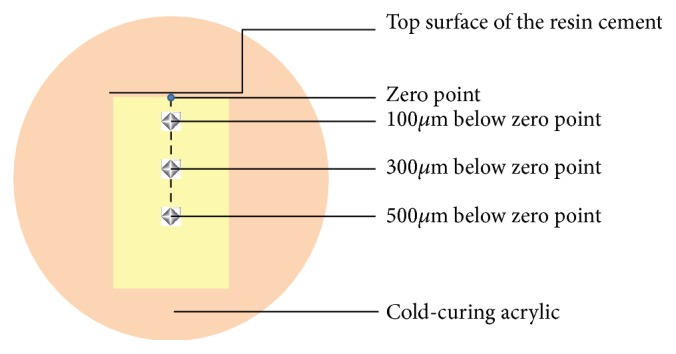
Schematic showing the determination of hardness indentation points on dual-cure resin cement horizontally embedded in acrylic.

The resin cement samples were longitudinally embedded in cold-curing acrylic (Meliodent, Bayer Dental, Newburg, Germany) in cylindrical molds (Figure 2). To prepare a smooth surface for VHN testing, the surfaces were subjected to wet polishing with 240-, 320-, 400-, 600-, and 1200-grit silicone carbide paper applied in a longitudinal direction. For microhardness measurements, the top surface of the resin cement facing the ceramic surface during the light exposure was marked as the zero point, and the Vickers hardness measurements were conducted at 100, 300, and 500 *μ*m below this zero point using a microhardness tester (402 MVD, Wolpert Wilson Instruments, Aachen, Germany) with a 50-g load applied for 15 s in the cross-sectional area. Three measurement depths were set by using the positioning knobs on the tester machine and the indentations were conducted by a pyramid-shaped microdiamond tip in order to give the hardness value (Figures 3 and 4). The depth and corner distance of the indentation left by the diamond tip was automatically measured by the software of the microhardness tester for obtaining the Vickers Hardness Number (VHN).

### 2.4. Statistical Analysis

VHNs obtained at different depths of dual-cure resin cement samples under different types and thicknesses of zirconia disks were statistically analyzed using three-way ANOVA in order to determine the effect of the zirconia type, specimen thickness, and measurement depth on VHN. The two-way interaction among groups was analyzed using independent sample t-tests, one-way ANOVA, and Bonferroni corrected post hoc tests (NCSS 2007, Kaysville, Utah, USA) (*α*=.05).

## 3. Results

Three-way ANOVA was carried out to find out the effect of independent variables (zirconia type, measurement depth, and zirconia thickness) on VHN of the resin cement. According to three-way ANOVA, the analysis model was significant, with an R^2^_adj_ of 0.840 (F: 66.465,* p<0.001*; Table 3). The analysis model showed that the main effect of the zirconia type, measurement depth, and zirconia thickness and all two-way interactions of these parameters were statistically significant, whereas three-way interaction of the parameters was insignificant (Table 3).

One-way ANOVA test showed that VHN of the dual-cure resin was significantly different between PRZ, PRA, and control groups at measurement depths of 100, 300, and 500 *μ*m (*p<0.001*, Table 4). Bonferroni corrected post hoc tests revealed that control group had the highest VHN values and the differences were significantly different between PRZ, PRA, and control groups (For 100-*μ*m* p<0.001, p<0.001, p=0.029*; for 300-*μ*m* p<0.001, p<0.001, p=0.034*; and for 500-*μ*m* p<0.001, p<0.001, p<0.001* respectively, data not shown) and VHN values of the dual-cure resin was significantly higher with PRA disks than with PRZ disks at measurement depths of 100, 300, and 500 *μ*m (*p=*0.018,* p*=0.003, and* p*<0.001, respectively data not shown). VHN showed a significant decrease with an increase in the measurement depth (*p*<0.001) for all groups polymerized under both PRA and PRZ discs whereas the difference was not significant for 100- and 300-*μ*m measurements of control group. For the groups polymerized beneath PRZ and PRA disks, VHN at a 100-*μ*m depth was significantly higher than that at 300- and 500-*μ*m depths, that at a 300-*μ*m depth was significantly higher than that at a 500-*μ*m depth (*p*<0.001; Table 4).

**Figure 5:**
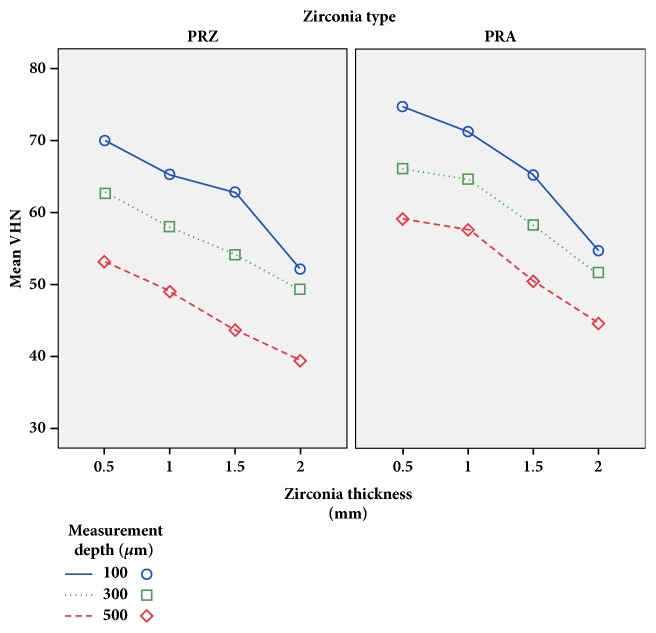
Mean Vickers hardness numbers (VHNs) for dual-cure resin according to the type and thickness of the overlying zirconia disk (PRZ: Prettau Zirconia; PRA: Prettau Anterior).

**Figure 6:**
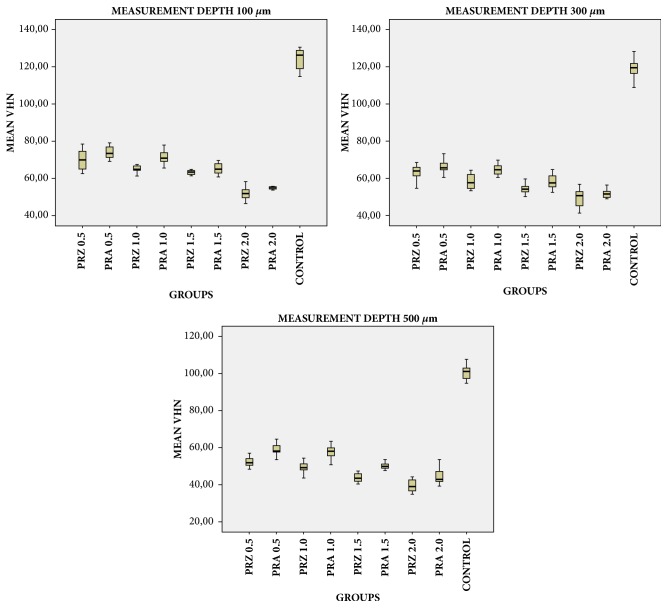
Error bar graphics of mean Vickers hardness numbers (VHNs) for all dual-cure resin cement groups at different measurement depths.

Different zirconia thicknesses led to significantly different VHNs. The mean VHN values significantly decreased with an increase in the zirconia thickness for both groups polymerized beneath PRA and PRZ (*p*<0.001; Table 5). VHN values of resin cement samples polymerized under PRA and PRZ disks with the same thicknesses were compared using independent samples t-tests (Table 5). At all thicknesses ignoring the measurement depth, mean VHN values were significantly higher for the groups polymerized under PRA disks than the groups polymerized under PRZ disks (*p*<0.05; Table 5). Pairwise intragroup comparisons according to different thicknesses were also performed; VHNs of resin cement samples decreased with an increase in the ceramic thickness (*p*<0.05; Table 5) whereas the difference was not statistically significant between 0.5 mm and 1.0 mm for both zirconia types (Table 5). Comparisons for both materials considering thickness and measurement depth are shown in Table 6 and schematically presented in Figures 5 and 6. For each measurement depth, resin cement hardness difference was statistically significant between two materials with the same thickness except PRZ_2.0_ and PRA_2.0_ (Table 6).

## 4. Discussion

In the present study, the microhardness of dual-cure resin cement cured underneath anterior and posterior monolithic zirconia disks with 0.5, 1.0, 1.5, and 2.0 mm thicknesses was investigated. The results showed that the null hypothesis tested in this study was rejected. In particular, microhardness of dual-cure resin cement was higher under anterior monolithic zirconia than posterior monolithic zirconia. Furthermore, increase in the thickness of the zirconia disks adversely affected the microhardness of the underlying resin cement.

Surface hardness which is identified as the material's resistance to indentation or penetration is accepted as one of the most crucial parameters for assessing physical properties of dental materials [14, 19]. It was previously reported that there is a strong relation between the microhardness of a resin and its conversion degree [14]. When the amount of cross-linked polymer increases the degree of conversion and relatedly the hardness of the material will be higher [14]. Therefore, microhardness measurement is usually preferred as a reliable technique for evaluating the conversion degree of resin-based luting cements [14, 19].

The results of the present study revealed that higher microhardness values were obtained for dual-cure resin samples polymerized under PRA samples than for those under PRZ samples for each zirconia thickness. An increase in the restoration's translucency enables higher visibility of the deepest layers, which allows the achievement of a much more natural appearance and also ensures required polymerization efficiency [20, 21]. An opaque restorative material would attenuate the curing light for the polymerization of the resin cement used for luting [22]. Sulaiman* et al*. [23] reported that different brands of partially stabilized monolithic zirconia show different translucency properties and fully stabilized monolithic zirconia is relatively more translucent than partially stabilized zirconia. In furtherance with the results of this study, current study showed that resin cement microhardness was higher when polymerized beneath fully stabilized PRA compared to partially stabilized PRZ material. The translucency of a dental ceramic is closely associated with its microstructure and chemical composition [11]. Muñoz et al. compared the crystalline phases of untreated Prettau® Anterior and Prettau Zirconia used in the current study after sintering using XRD analysis, and reported a higher weight percentage of cubic phase in Prettau® Anterior compared to Prettau Zirconia [11]. According to the results of the current study, it can be stated that higher cubic phase in PRA group resulted in an improved translucency, thus increasing the polymerization efficiency of the resin cement samples. Further research is suggested in order to evaluate the relationship between crystalline phases, translucency, and polymerization efficiency of resin cements used beneath different zirconia types.

In accordance with the previous study results, the present study revealed that for both monolithic zirconia types resin cement hardness decreased as the thickness of the zirconia material increased [11, 23]. A previous study reported that different brands of partially stabilized monolithic zirconia show different translucency properties that are mostly affected by the material thickness [23]. Minimum material thickness for monolithic zirconia restorations is reported to be 0.5 mm but in case of fabricating anatomical posterior restorations with monolithic zirconia the thickness can reach to 2.0 mm [24]. The findings of this study showed that the difference between microhardness values of dual-cure resin cement were statistically significant except the groups PRZ_2.0_ and PRA_2.0_ for all measurement depths. Although dual-cure resin materials undergo both types of polymerization, Panavia F 2.0 mostly depends on light irradiation and polymerization may not be complete without sufficient light irradiation [16, 25–28]. For this reason, the use of Panavia F 2.0 should be preferred in cases where the polymerizing light can reach the light-activated paste, such as anterior and posterior monolithic zirconia restorations ≤ 1.5 mm thickness.

It can be considered that when the restoration thickness is ≥ 2.0 mm for anterior and posterior monolithic zirconia materials, the material difference may become insignificant because of the inadequate light activation which may be related to decreased translucency properties. Therefore, it may be recommended to use extended light curing, dual-cured resin cements with a higher chemically curing component than Panavia F 2.0, or a self-cure resin cement for anterior and posterior monolithic zirconia restorations with ≥ 2.0 mm thickness.

Previously it was reported that microhardness of resin cement is also influenced by the shade of the cement itself [14, 29, 30]. The hardness of the resin was found to decrease as the shade of the cement gets darker in case of direct light exposure [14, 29, 30]. Moreno et al. recently evaluated the cement shade effect on microhardness of dual-cure resin cement indirectly light-activated beneath different ceramics and in accordance with the previous study results, they have reported that a specific activation strategy is essential for each cement shade in order to maximize the material hardness [31]. Transparent resin cements enable higher depth of cure and microhardness values due to their capability of absorbing more light than the opaque cements [14, 30–32]. Therefore, the manufacturers recommend translucent shades for the cementation of the restorations made of metal-oxide ceramics and increasing irradiation time of the resin cement may result in higher hardness values as the shade of the cement gets darker [32]. In the present study, the ‘light (translucent) shade' kit of Panavia F 2.0 was chosen in order to minimize the risk of having an impact on cement microhardness evaluation and focus on the influence of zirconia type and thickness on resin cement polymerization efficiency. Nonetheless, the clinicians should mind that resin cements may have decreased hardness in case of opaque shade choice in order to mask the dark abutment tooth shades and increasing the exposure duration may help to increase the opaquer and/or darker shaded resin cement microhardness especially for fine anterior monolithic zirconia-based restorations.

In the present study, flat zirconia disks were used for resin cement polymerization enabled placement of the light tip in direct contact with the ceramic surface. In clinical situations, Prettau® Anterior blocks are mostly used for anterior restorations, which have relatively flat surfaces. Therefore, dual-cure resin cements can be polymerized by placing the light tip in direct contact with the restoration. In case of posterior restorations fabricated from Prettau material, occlusal cusps may inhibit the placement of the light tip further from the cement, which may have a negative effect on resin cement polymerization [33, 34]. Therefore, the clinicians should be aware that investigated resin cement may have lower hardness values beneath anatomical crowns than experimental specimens especially for posterior monolithic zirconia.

A major limitation of this study is the small number of samples in each group because of restricted clinical circumstances. Only one brand of each material (anterior monolithic zirconia, posterior monolithic zirconia, and resin cement) was used in order to keep the focus on the difference between anterior and posterior monolithic zirconia. However, there are more factors that could affect the polymerization of the resin cement cured beneath zirconia-based restorations. In order to develop clinical recommendations, further studies are needed to confirm our results through comparisons of monolithic zirconia materials with different brands, compositions, and shades when luted using resin cements with differing ratios of chemical and light-activated components.

Within the limitations of the study, the following conclusions can be drawn: microhardness values of dual-cure resin cement samples polymerized beneath PRA based materials were higher than that of resin cement samples polymerized under PRZ based materials for the same material thickness and measurement depth. Increase in zirconia thickness leads to significantly lower microhardness values for the underlying resin cement for both PRA and PRZ materials. Clinicians should consider that an increase in the thickness of both PRA and PRZ based restorations could result in insufficient light transmission, which affects the long-term durability of the resin cement and restoration. On the basis of this finding, clinicians can select a better material for each given situation, and they should also consider the efficiency of light curing under thicker zirconia restorations.

## Figures and Tables

**Table 1:** Monolithic zirconia types used in the study as disclosed by the manufacturer.

**Brand name /Indication**	**Stability**	**Abbreviation **	**Manufacturer**	**Composition** **∗**
Prettau Anterior/anterior restorations	Fully stabilized	PRA	Zirkonzahn GmbH, Bruneck, Italy	8-12% Y_2_O_3_, 0-1% Al_2_O_3_, max. 0.02% SiO_2_, max. 0.01 Fe_2_O_3_, max. 0.04% Na_2_O

Prettau Zirconia/posterior restorations	Partially stabilized	PRZ	Zirkonzahn GmbH, Bruneck, Italy	4-6% Y_2_O_3_, 0-1% Al_2_O_3_, max. 0.02% SiO_2_, max. 0.01 Fe_2_O_3_, max. 0.04% Na_2_O

**Table 2:** Study group abbreviations.

**Groups**	**Zirconia thickness (mm)**
PRZ_0.5_ - PRA_0.5_	0.5
PRZ_1.0_ – PRA_1.0_	1.0
PRZ_1.5_ – PRA_1.5_	1.5
PRZ_2.0_ – PRA_2.0_	2.0

PRZ: Prettau Zirconia; PRA: Prettau Anterior.

**Table 3:** Findings of three-way ANOVA to determine the effects of zirconia type, measurement depth, and zirconia specimen thickness on the Vickers hardness number (VHN) for dual-cure resin cement under PRA and PRZ zirconia disks.

**Source of variation**	**Sum of squares**	**df**	**Mean square**	**F**	***p***
**Intercept**	948546.511	1	948546.511	63040.071	**<0.001** **∗**
**Zirconia type**	1699.445	1	1699.445	112.945	**<0.001** **∗**
**Depth**	10724.956	2	5362.478	356.388	**<0.001** **∗**
**Thickness**	10033.696	3	3344.565	222.279	**<0.001** **∗**
**Zirconia type ** **∗** ** Depth**	106.596	2	53.298	3.542	**0.030** **∗**
**Zirconia type ** **∗** ** Thickness**	129.221	3	43.074	2.863	**0.037** **∗**
**Depth ** **∗** ** Thickness **	287.045	6	47.841	3.179	**0.005** **∗**
**Zirconia type ** **∗** ** Depth ** **∗** ** Thickness**	20.895	6	3.483	.231	**0.966**

*Adjusted R* ^*2*^ *, 0.840.    ∗p<0.05.*

**Table 4:** Mean Vickers hardness numbers for dual-cure resin cement at different measurement depths indirectly irradiated under PRZ and PRA zirconia disks and directly light activated control group.

**Measurement ** **Depth (*μ*m)**	**Zirconia type**		**Control**	^**a**^ ***p***
	**PRZ**	**PRA**		
	**Mean±SD**	**Mean±SD**	**Mean±SD**	
**100**	62.53±7.53	66.46±8.45	124.22±5.42	***<0.001*** **∗**
**300**	56.04±6.40	60.11±6.55	119.20±5.77	***<0.001*** **∗**
**500**	46.31±7.01	52.88±6.81	100.43±3.75	***<0.001*** **∗**

^**a**^ ***p***	**<0.001** **∗**	**<0.001** **∗**	** <0.001** **∗**	

^‡^ **100 vs 300**	**<0.001** **∗**	**<0.001** **∗**	** 0.051**	
^‡^ **100 vs 500**	**<0.001** **∗**	**<0.001** **∗**	** <0.001** **∗**	
^‡^ **300 vs 500**	**<0.001** **∗**	**<0.001** **∗**	** <0.001** **∗**	

^a^One-way ANOVA.  ^‡^Bonferroni-corrected *p*-values.

PRZ: Prettau Zirconia; PRA: Prettau Anterior.

*∗p*<0.001.

**Table 5:** Mean Vickers hardness numbers for dual-cure resin cement depending on the thickness of the overlying zirconia disk.

**Thickness ** **(mm)**	**Zirconia type**		^**a**^ ***p***
	**PRZ**	**PRA**	
	**Mean±SD**	**Mean±SD**	
**0.5**	61.93±9.11	66.60±7.53	***0.021*** **∗**
**1.0**	57.43±7.60	64.44±6.55	***<0.001*** **∗**
**1.5**	53.53±8.37	57.94±6.80	***0.017*** **∗**
**2.0**	46.95±6.72	50.30±5.48	***0.024*** **∗**

^**b**^ ***p***	**<0.001** **∗**	**<0.001** **∗**	

^‡^ **0.5 vs. 1.0**	**0.110**	**0.999**	
^‡^ **0.5 vs. 1.5**	**<0.001** **∗**	**<0.001** **∗**	
^‡^ **0.5 vs. 2.0**	**<0.001** **∗**	**<0.001** **∗**	
^‡^ **1.0 vs. 1.5**	**<0.001** **∗**	**<0.001** **∗**	
^‡^ **1.0 vs. 2.0**	**<0.001** **∗**	**<0.001** **∗**	
^‡^ **1.5 vs. 2.0**	**0.004** **∗**	**<0.001** **∗**	

^a^Independent samples t-test.   ^b^One-way ANOVA.   ^‡^Bonferroni-corrected *p*-values.

PRZ: Prettau Zirconia; PRA: Prettau Anterior.

*∗p*<0.05.

**Table 6:** Independent samples t-test and one-way ANOVA results for all measurements. Means followed by different capital letters in the same line and different small letters in the same column were statistically different at p<0.05.

**Zirconia ** **thickness**	**Zirconia type**		**Measurement depth**	
		**100 *µ*m**	**300 *µ*m**	**500 *µ*m**
	**Mean±SD**	**Mean±SD**		**Mean±SD**
**0.5 mm**	PRZ	69.95±5.35 **c,A**	62.67±4.70 **c,B**	53.15±7.64 **c,C**
	PRA	74.69±5.09 **b,A**	66.06±3.25 **b,B**	59.03±3.15 **b,C**
**1 mm**	PRZ	65.26±2.55 **d,A**	58.00±4.14 **d,B**	49.00±3.89 **d,C**
	PRA	71.22±3.28 **c,A**	64.58±3.04 **c,B**	57.51±3.72 **b,C**
**1.5 mm**	PRZ	62.80±2.83 **e,A**	54.15±2.73 **e,B**	43.64±2.52 **e,C**
	PRA	65.22±2.96 **d,A**	58.16±3.68 **d,B**	50.42±2.25 **d,C**
**2 mm**	PRZ	52.10±3.47 **f,A**	49.33±4.82 **f,A**	39.41±3.37 **e,B**
	PRA	54.69±2.79 **f,A**	51.64±2.60 **f,A**	42.55±4.69 **e,B**

**Control**		124.22±5.42 **a,A**	119.20±5.77 **a,A**	100.43±3.75 **a,B**

## Data Availability

The data used to support the findings of this study are available from the corresponding author upon request.
